# Automatic Change Detection to Facial Expressions in Adolescents: Evidence from Visual Mismatch Negativity Responses

**DOI:** 10.3389/fpsyg.2016.00462

**Published:** 2016-03-30

**Authors:** Tongran Liu, Tong Xiao, Jiannong Shi

**Affiliations:** ^1^Key Laboratory of Behavioral Science, Institute of Psychology, Chinese Academy of SciencesBeijing, China; ^2^Natural Language Processing Laboratory, College of Information Science and Engineering, Northeastern UniversityLiaoning, China; ^3^Department of Learning and Philosophy, Aalborg UniversityAalborg, Denmark

**Keywords:** automatic change detection, ERPs, visual mismatch negativity, facial expression perception, adolescence

## Abstract

Adolescence is a critical period for the neurodevelopment of social-emotional processing, wherein the automatic detection of changes in facial expressions is crucial for the development of interpersonal communication. Two groups of participants (an adolescent group and an adult group) were recruited to complete an emotional oddball task featuring on happy and one fearful condition. The measurement of event-related potential was carried out via electroencephalography and electrooculography recording, to detect visual mismatch negativity (vMMN) with regard to the automatic detection of changes in facial expressions between the two age groups. The current findings demonstrated that the adolescent group featured more negative vMMN amplitudes than the adult group in the fronto-central region during the 120–200 ms interval. During the time window of 370–450 ms, only the adult group showed better automatic processing on fearful faces than happy faces. The present study indicated that adolescent’s posses stronger automatic detection of changes in emotional expression relative to adults, and sheds light on the neurodevelopment of automatic processes concerning social-emotional information.

## Introduction

From a neurodevelopmental perspective, adolescence is a crucial period for cognitive and emotional development (Paus, 2005; Steinberg, 2005; Yurgelun-Todd, 2007; Casey et al., 2008; Ahmed et al., 2015). During adolescence, dramatic changes in white and gray matter density (Giedd et al., 1999) reflect alterations in the neural plasticity of regions supporting social and cognitive functions (Steinberg, 2005; Blakemore and Choudhury, 2006; Casey et al., 2010, 2011; Somerville et al., 2010; Burnett et al., 2011; Pfeifer et al., 2011; Kadosh et al., 2013). In particular, the adolescent brain appears to be more emotionally driven, featuring heightened sensitivity to affective information and a subsequent increase in vulnerability to affective disorders (Monk et al., 2003; Dahl, 2004; Taylor et al., 2004; Scherf et al., 2011). This vulnerability likely reflects the high risk for mood and behavioral disorders observed during adolescence (Nelson et al., 2005; Blakemore, 2008; Casey et al., 2008). Therefore, it is vital to investigate the neurodevelopmental processes underlying emotional development in healthy adolescents, and identify appropriate standards to characterize abnormal development in adolescents at risk of mood disorders and psychosis (Passarotti et al., 2009; van Rijn et al., 2011).

Facial expressions encode essential social-emotional information, wherein the appropriate perception and interpretation of facial expression is important for the development and maturation of social-emotional processing in adolescence (Monk et al., 2003; Herba and Phillips, 2004; Taylor et al., 2004; Guyer et al., 2008; Hare et al., 2008; Passarotti et al., 2009; Segalowitz et al., 2010; Scherf et al., 2012; van den Bulk et al., 2012). Furthermore, electrophysiological studies indicate that several event-related potential (ERP) components are associated with different stages of facial expression processing. This includes the P1 component, which peaks at approximately 100 ms after stimulus onset, and mediates the early visual processing of face categorization in the temporal and occipital areas (Linkenkaer-Hansen et al., 1998; Itier and Taylor, 2002; Pourtois et al., 2005). In addition, the N170 component has been reported to reflect the structural encoding of faces and/or facial expression within the temporal-occipital region (Bentin et al., 1996; Rossion et al., 1999a,b; Taylor et al., 1999; Itier and Taylor, 2002, 2004a,b; Segalowitz et al., 2010; Zhang et al., 2013; Dundas et al., 2014). Moreover, the late positive potential (LPP), which peaks approximately 200 ms after exposure to emotional stimuli, mediates the sustained attention to affective information (Cuthbert et al., 2000; Itier and Taylor, 2002; Olofsson et al., 2008; Foti et al., 2009; Hajcak and Dennis, 2009; Hajcak et al., 2011). Accordingly, LPP amplitudes are greater in response to emotional rather than neutral stimuli (Kujawa et al., 2012a,b).

In electrophysiological studies, children and adolescents demonstrate longer N170 latencies and smaller negative N170 amplitudes compared to adults (Taylor et al., 1999; Dundas et al., 2014). Furthermore, adolescents exhibit greater LPP amplitudes at occipital sites relative to young adults (Gao et al., 2010). Neuroimaging studies indicate that the neural networks implicated in the perception of facial expression, including the limbic, amygdala, temporal, parietal and prefrontal regions, undergo a period of maturation between early childhood and late adolescence (Taylor et al., 1999; Killgore et al., 2001; Monk et al., 2003; McClure et al., 2004; Nelson et al., 2005; Batty and Taylor, 2006; Lobaugh et al., 2006; Costafreda et al., 2008). However, the majority of existing studies have investigated the role of attentional focus in the perception of facial expression, while the automatic change detection of changes in emotional expression has received relatively less attention. Since this process is also crucial for the development of social-emotional communication in adolescence (Miki et al., 2011; Hung et al., 2012), it requires a stronger focus in current research.

Electrophysiological studies have demonstrated that the ERP component mismatch negativity (MMN) reflects automatic change detection of auditory and visual stimuli (auditory MMN [aMMN] and visual MMN [vMMN], respectively) (Czigler, 2007; Näätänen et al., 2007). In the passive oddball paradigm, standard stimuli, which are presented at frequent intervals, are randomly replaced by deviant stimuli. The MMN value is therefore computed by subtracting the neural responses to standard stimuli from that of deviant stimuli (Pazo-Alvarez et al., 2003; Czigler, 2007; Näätänen et al., 2007). vMMN responses to facial expressions have been identified between 70 and 430 ms post-stimulus presentation within single (Susac et al., 2004; Zhao and Li, 2006; Gayle et al., 2012) or multiple time windows (Astikainen and Hietanen, 2009; Chang et al., 2010; Kimura et al., 2012; Li et al., 2012; Stefanics et al., 2012; Astikainen et al., 2013; Kecskés-Kovács et al., 2013; Kreegipuu et al., 2013). Moreover, the neural distribution of emotion-related vMMN has been reported to include the bilateral posterior occipito-temporal regions (Susac et al., 2004; Zhao and Li, 2006; Astikainen and Hietanen, 2009; Chang et al., 2010; Gayle et al., 2012; Kimura et al., 2012; Li et al., 2012; Stefanics et al., 2012; Astikainen et al., 2013; Kreegipuu et al., 2013) and the anterior frontal regions (Astikainen and Hietanen, 2009; Kimura et al., 2012; Stefanics et al., 2012). The methodology of expression-related vMMN studies was improved by Stefanics et al. (2012), wherein a primary task was added to the emotional oddball paradigm. The primary task aimed to draw the participant’s attention toward a central cross, and the participant was required to press a response button when the central cross changed in size, and ignore the bilateral emotional faces. The emotion oddball paradigm was presented simultaneously with the primary task, but the changes in expressions occurred independent to the changes in the size of the central cross. The authors reported significant expression-related vMMN at the bilateral occipito-temporal electrode sites, 170–360 ms following stimulus presentation (Stefanics et al., 2012). Based on prior vMMN studies, the emotional vMMN component might provide an important measure of automatic emotional processing in adolescence, and a means of further exploring the automatic detection of regularity violation, and predictive memory representation of facial expressions.

The main aim of the current study was to investigate the automatic detection of changes in facial expression in adolescents, which was achieved using an oddball paradigm similar to that previously described. Expression-related vMMN responses were then analyzed and compared between two age groups (adolescent and adult) to reveal the differing neural dynamics in the automatic detection of changes in facial expression.

## Materials and Methods

### Subjects

The present study included 36 subjects, including 19 adolescent participants (male, 9, female, 10; age range 14.2–14.9 years; mean age, 14.6 years) recruited from a normal middle school, and 17 adult participants (male, 8, female, 9; age from 21.3 to 29.7 years; mean age, 26.2 years) recruited as undergraduate students. The enrolment of participants occurred in agreement with the Declaration of Helsinki. This study was approved by the Ethics Committee of the Institute of Psychology, Chinese Academy of Sciences. Written informed consent was obtained from both participants and the parents of adolescent participants. Each subject was paid 100 RMB for participating. All participants were right handed with normal or corrected-to-normal visual acuity, and none had been diagnosed with neurological or psychiatric disorders. All participants were naïve to the purposes of the experiment.

### Materials and Procedure

**FIGURE 1:**
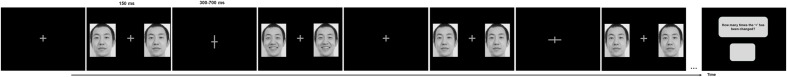
**The sample of experimental procedure in the happy oddball condition.** A central cross changed (wider or longer) occasionally during each sequence, and participants were instructed to detect the changes and to report how many times the cross had been changed at the end of each sequence. Two identical faces with the same expression were displayed on the bilateral sides of the cross for 150 ms, followed by the inter-stimulus interval of 300–700 ms. The presentation of face-pairs and the changes of the fixation cross were independent.

**Figure 1** illustrates the procedure used in the present study. The presentation screen was a computer monitor (17′, 1024 × 768 at 100 Hz) with a black background, and participant’s viewing distance was 60 cm. A primary task (in the central visual field) and an expression-related oddball task (on the bilateral sides of the central visual field) were presented simultaneously but independently on the screen. Participants were required to focus their attention on the primary task and ignore the surrounding emotional stimuli (images of facial expressions). This design was used to ensure that the perception of emotional information (presented in the oddball paradigm) was automatic.

The emotional stimuli used were images of facial expressions (in a light gray color) from 10 Chinese models (male, 5; female, 5) in three set expressions: neutral, happy, and fearful. Normative 9-point scale ratings were carried by another 30 volunteers (male, 16, female, 14; age range 22.3-28.7 years) to assess the valence and arousal of each facial image. For the valence rating, a *t*-test demonstrated that happy images (*M* = 7.84, *SD* = 0.23) featured higher scores than neutral images (*M* = 5.03, *SD* = 0.21), while neutral images featured higher scores than fearful images (*M* = 1.92, *SD* = 0.2) (*ps* < 0.001). The reliability of the volunteers’ judgments with regard to valence was high (Kendall’s *W* = 0.94, χ^2^ = 816.91, *p* < 0.001). For the arousal rating, happy (*M* = 6.8, *SD* = 0.21) and fearful images (*M* = 6.97, *SD* = 0.26) produced a higher level of arousal than neutral images (*M* = 1.33, *SD* = 0.25) (*ps* < 0.001), while no significant differences were observed between happy and fearful images with regard to arousal scores (*p* > 0.05). The reliability of the volunteers’ judgment of arousal ratings was high (Kendall’s *W* = 0.76, χ^2^ = 663.48, *p* < 0.001).

The face-pairs that were presented in each sequence were randomly selected from the stimulus pool of 10 models. For each trial of the oddball task, two identical expressions from one model were synchronously displayed on both sides of the central cross. Each face was displayed with a visual angle of 6° horizontally and 8° vertically. Each face-pair was displayed for 150 ms, followed by an inter-stimulus interval (offset-to-onset) of 300–700 ms. Two experimental conditions were adopted: one happy oddball condition and one fearful oddball condition, and the presentation order of the two oddball conditions was randomized across participants. For the happy oddball condition, happy faces were presented as the deviant stimuli (probability of 0.2) and neutral faces as the standard stimuli (probability of 0.8). For the fearful oddball condition, fearful faces were presented as the deviant stimuli (probability of 0.2) and neutral expressions as the standard stimuli (probability of 0.8). Each condition consisted of six practice sequences and 60 formal sequences, and each sequence contained 10 face-pair presentations (two deviant stimuli and eight standard stimuli). In total, 480 standard stimuli and 120 deviant stimuli were presented for each condition. Furthermore, each deviant was presented after at least two standards in each sequence, and the average position of deviant was 7.2. Each sequence lasted for 10 s, and the next sequence started directly after participants reported the change number. These settings guaranteed that memory trace could survive the break and vMMN responses could be elicited (Sulykos et al., 2013).

For the primary task, participants were instructed to detect and count how many times the central cross (“+”) had changed in size (the horizontal line of the cross was longer than its vertical line, or the length of the vertical line was wider), and to report this number at the end of each sequence. The start and end of each sequence was synchronized with that of the oddball task. Each change in cross size lasted 300 ms, before the cross returned to its original size. During each sequence, there were nine possible options for the number of cross changes. Participants were required to concentrate on counting the number of changes, and to press the corresponding button on the keyboard (“0” to “9”) with their right index finger at the end of each sequence. The answer screen did not fade away until participants pressed a button.

### EEG Recording and Analysis

Sixty-four electrodes embedded in a NeuroScan Quik-Cap were used to record electroencephalography (EEG) data, and the electrodes were positioned according to the 10–20 system locations. EEG data were collected and analyzed, using the nose as reference. For electrooculography (EOG) recording, four bipolar electrodes were positioned on the inferior and superior regions of the left eye and the outer canthi of both eyes to monitor vertical and horizontal EOG (VEOG and HEOG). Electrode impedance was maintained below 5 kΩ, and EEG signal was continuously recorded with online band-pass filters at 0.05–100 Hz with a nose reference. The signal was amplified using SynAmps amplifiers with a sample rate of 500 Hz. The signal was edited to include 50 ms prior to (for baseline correction) and 450 ms after stimulus onset. Epochs contaminated by eye blinks, movement artifacts, or muscle potentials exceeding ±70 μV at any electrode were excluded. ERPs underwent offline Zero Phase Shift digital filtering (bandwidth: 1–30 Hz, slope: 24 dB/octave).

For the fearful condition, the mean number of trials for the deviant condition (fearful expression) was 94 for adolescents and 97 trials for adults, while a mean of 323 trials was calculated for the standard condition (neutral expression) in adolescents and 328 in adults. For the happy condition, the mean number of trials for the deviant condition (happy expression) was 94 for adolescents and 96 in adults, while a mean of 325 trials were calculated for the standard condition (neutral expression) in adolescents and 324 in adults.

Three ERP components were analyzed: P1 (100–170 ms), N170 (150–220 ms) and LPP (210–280 ms) within the temporal and occipital regions (average neural activation of the electrodes positioned at TP7, P7, PO7, CB1, O1, TP8, P8, PO8, CB2, O2) (Bentin et al., 1996; Campanella et al., 2002; Pourtois et al., 2005). A three-way repeated ANOVAs was used to analyze the peak latency and amplitude of each ERP component, with the independent variables of Age-group (adolescent vs. adult), Stimulus-type (standard vs. deviant), and Expression condition (happy vs. fearful). Greenhouse–Geisser corrections for violations of sphericity were used where appropriate and significant interactions were further investigated using SIDAK *post hoc* tests with Bonferroni correction for multiple comparisons.

The difference waveforms were created by subtracting the ERP responses to standard stimuli from the ERP responses to deviant stimuli, to analyze the automatic processing of emotional stimuli (Czigler, 2007; Näätänen et al., 2007). The time window of vMMN was determined according to previous vMMN literature and the visual inspection of current waveforms (Stefanics et al., 2014, 2015). Three time windows were selected for vMMN analysis: 120–200 ms, 230–320 ms, and 370–450 ms within the fronto-central (averaging the electrodes at F1, FC1, C1 F2, FC2, and C2) and occipito-temporal areas (averaging the electrodes at TP7, P7, PO7, O1, TP8, P8, PO8, and O2). The mean amplitudes of difference waveform responses were compared with zero to confirm that the vMMN responses were significant for different brain regions and time windows with regard to both age groups. Next, a three-way repeated ANOVA was used to analyze the peak amplitudes of vMMNs with regard to the independent variables of Age-group (adolescent vs. adult), Expression condition (happy vs. fearful), and Anterior-posterior distribution (fronto-central vs. occipito-temporal). Greenhouse–Geisser corrections for violations of sphericity were applied where appropriate, and significant interactions were further analyzed using the SIDAK test.

## Results

### Effect of Deviant and Standard Emotional Stimuli on ERPs

**FIGURE 2:**
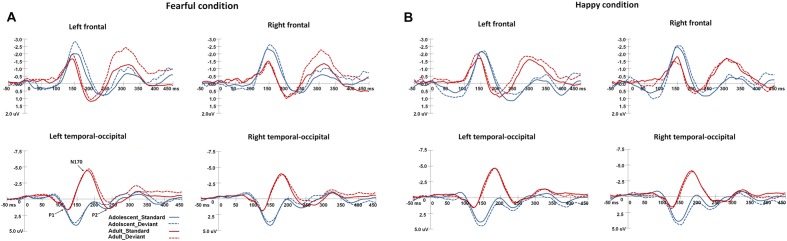
**The grand-average ERPs elicited by the standard and deviant stimuli in the happy and fearful oddball conditions for both adolescent and adult groups.** ERP responses in the electrodes of F1, FC1, and C1 were averaged to plot the neural activity over the left frontal areas, and F2, FC3, and C2 were averaged for the right frontal areas. The ERP responses in the left occipito-temporal areas were averaged from the electrodes of TP7, P7, PO7, CB1, and O1, and the ERP responses in the electrodes of TP8, P8, PO8, CB2, and O2 were averaged for the right occipito-temporal areas. **(A)** Shows the ERP waveforms in the fearful condition, and **(B)** for the ERP waveforms in the happy condition.

**Figure 2** shows the grand-averaged ERPs waveforms elicited by standard and deviant stimuli in both the happy and fearful oddball conditions.

For P1 latency, the main effect of Age-group was significant [*F*(1,34) = 64.1, *p* < 0.001, η^2^ = 0.62], and adolescents demonstrated longer P1 latencies than adults (145 ms [*SD* = 2 ms] vs. 120 ms [*SD* = 2.4 ms]). The main effect of Expression condition was also significant [*F*(1,34) = 4.2, *p* < 0.05, η^2^ = 0.1], and participants demonstrated faster P1 responses in the happy condition compared to the fearful (132 ms [*SD* = 1.7 ms] vs. 134 ms [*SD* = 1.5 ms]). With regard to P1 amplitudes, the main effect of Age-group was significant [*F*(1,34) = 10.6, *p* < 0.005, η^2^ = 0.2], wherein adolescents featured larger P1 amplitudes than adults (4.4 μV [*SD* = 0.5 μV] vs. 1.9 μV [*SD* = 0.6 μV]). The interaction effect of Stimulus-type × Expression condition [*F*(1,34) = 4.1, *p* < 0.05, η^2^ = 0.1], was also significant, wherein deviant stimuli induced larger P1 amplitudes than standard stimuli in the happy condition (3.4 μV [*SD* = 0.38 μV] vs. 2.9 μV [*SD* = 0.38 μV], *p* < 0.05).

For N170 latency, the main effect of Age-group was significant [*F*(1,34) = 25.4, *p* < 0.001, η^2^ = 0.4], and adolescents featured longer N170 latencies than adults (199 ms [*SD* = 2.1 ms] vs. 182 ms [*SD* = 2.5 ms]). With regard to N170 amplitudes, the main effect of Age-group was also significant [*F*(1,34) = 15.8, *p* < 0.001, η^2^ = 0.29], and adolescents demonstrated less negative N170 amplitudes than adults (-1.1 μV [*SD* = 0.6 μV] vs. -4.6 μV [*SD* = 0.7 μV]).

For LPP latency, the main effect of Age-group was significant [*F*(1,34) = 12.8, *p* < 0.005, η^2^ = 0.25], and adolescents had faster LPP responses than adults (238 ms [*SD* = 2.7 ms] vs. 253 ms [*SD* = 3.2 ms]). The interaction effect of Expression condition × Age-group was particularly significant [*F*(1,34) = 3.8, *p* < 0.05, η^2^ = 0.1], whereupon *post hoc* analysis indicated that adults had faster LPP responses in the fearful condition than in the happy condition (250 ms [*SD* = 3.5 ms] vs. 256 ms [*SD* = 3.4 ms], *p* < 0.05). For LPP amplitudes, the interaction effect of Expression condition × Stimulus-type was significant [*F*(1,34) = 12.6, *p* < 0.005, η^2^ = 0.25], and *post hoc* analysis showed that the deviant stimuli elicited larger LPP amplitudes in the happy condition than that in the fearful condition (1.9 μV [*SD* = 0.4 μV] vs. 1.2 μV [*SD* = 0.5 μV], *p* < 0.05). In addition, the standard stimuli induced larger LPP amplitudes than the deviant stimuli in the fearful condition (1.7 μV [*SD* = 0.4 μV] vs. 1.2 μV [*SD* = 0.5 μV], *p* < 0.05), however, the standard stimuli elicited smaller LPP amplitudes than deviant stimuli in the happy condition (1.5 μV [*SD* = 0.5 μV] vs. 1.9 μV [*SD* = 0.4 μV], *p* < 0.05).

### vMMN Responses in the Happy and Fearful Oddball Conditions

**FIGURE 3:**
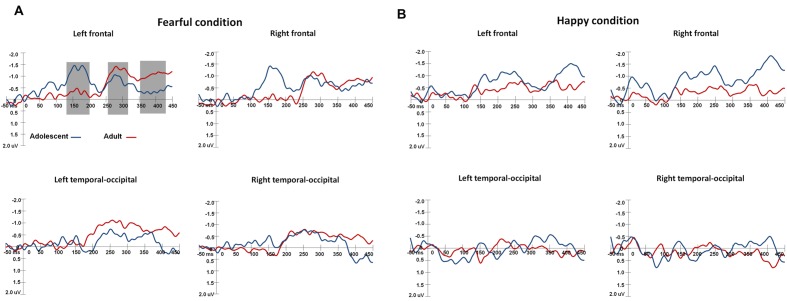
**The waveforms of vMMNs in both happy and fearful conditions.** The ERP responses in the electrodes of F1, FC1, and C1 were averaged to plot the neural activity over the left frontal areas, and F2, FC3, and C2 were averaged for the right frontal areas. The ERP responses in the electrodes of TP7, P7, PO7, CB1, and O1 were averaged to plot the neural activity over the left occipito-temporal areas, and TP8, P8, PO8, CB2, and O2 averaged for the right occipito-temporal areas. **(A)** Shows the vMMN waveforms in the fearful condition, and **(B)** for the vMMN waveforms in the happy condition.

**FIGURE 4:**
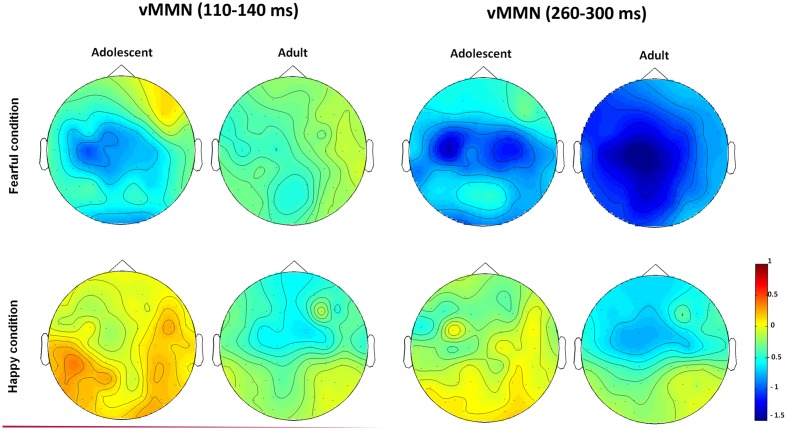
**The topographic maps of the vMMNs for both adolescent and adult groups in the happy and fearful conditions**.

The waveforms of vMMNs in both the happy and fearful conditions are displayed in **Figure 3** and the topographic maps are presented in **Figure 4**. The mean amplitudes of difference waveforms were compared with zero, and the *t*-tests results indicated that the vMMN components were significant for all time windows, brain regions, and emotional conditions for adults and adolescents (see **Table 1**).

**Table 1:** The comparisons between mean amplitudes (μV) of VMMN and zero in different brain areas, time windows, and emotional conditions.

Group	Emotional condition	Time windows	Brain areas	Mean (*SD*)	Significance
Adolescent	Fearful	100–200 ms	Fronto-central	-2.2 (1.58)	*t* = -6.05, *p* < 0.001
			Occipito-temporal	-1.56 (1.52)	*t* = -4.46, *p* < 0.001
		230–320 ms	Fronto-central	-2.52 (1.3)	*t* = -8.46, *p* < 0.001
			Occipito-temporal	-1.93 (1.33)	*t* = -6.32, *p* < 0.001
		370–450 ms	Fronto-central	-1.86 (2.03)	*t* = -6.18, *p* < 0.001
			Occipito-temporal	-1.20 (1.69)	*t* = -4.27, *p* < 0.001
	Happy	100–200 ms	Fronto-central	-1.79 (0.76)	*t* = -10.23, *p* < 0.001
			Occipito-temporal	-0.87 (0.91)	*t* = -4.18, *p* < 0.005
		230–320 ms	Fronto-central	-2.11 (1.14)	*t* = -8.08, *p* < 0.001
			Occipito-temporal	-1.06 (1.14)	*t* = -4.04, *p* < 0.005
		370–450 ms	Fronto-central	-2.14 (1.46)	*t* = -7.08, *p* < 0.001
			Occipito-temporal	-1.17 (0.94)	*t* = -5.27, *p* < 0.001
Adult	Fearful	100–200 ms	Fronto-central	-1.17 (1.26)	*t* = -3.83, *p* < 0.005
			Occipito-temporal	-1.12 (1.06)	*t* = -4.36, *p* < 0.001
		230–320 ms	Fronto-central	-2 (1.47)	*t* = -5.6, *p* < 0.001
			Occipito-temporal	-1.74 (1.16)	*t* = -6.15, *p* < 0.001
		370–450 ms	Fronto-central	-1.89 (1.32)	*t* = -5.89, *p* < 0.001
			Occipito-temporal	-1.52 (1.21)	*t* = -5.16, *p* < 0.001
	Happy	100–200 ms	Fronto-central	-1.39 (1.44)	*t* = -3.97, *p* < 0.005
			Occipito-temporal	-1.07 (1.48)	*t* = -2.98, *p* < 0.01
		230–320 ms	Fronto-central	-1.61 (1.7)	*t* = -3.91, *p* < 0.005
			Occipito-temporal	-1.06 (1.57)	*t* = -2.78, *p* < 0.05
		370–450 ms	Fronto-central	-1.37 (1.88)	*t* = -3.01, *p* < 0.01
			Occipito-temporal	-0.54 (1.99)	*t* = -2.12, *p* < 0.05


For early vMMN values (120–200 ms), the interaction between Age-group and Anterior-posterior distribution was significant [*F*(1,34) = 4.97, *p* < 0.05, η^2^ = 0.13], and the adolescent group demonstrated more negative vMMN amplitudes than the adult group over the fronto-central regions (*p* < 0.05). vMMN amplitude was more negative in the fronto-central regions than occipito-temporal areas in the adolescent group (*p* < 0.001), but not the adult group (*p* = 0.33).

For middle vMMN values at the 230–320 ms interval, the main effect of Expression condition was significant [*F*(1,34) = 9.41, *p* < 0.005, η^2^ = 0.22], while vMMNs was greater in the fearful condition relative to the happy condition. The main effect of Anterior-posterior distribution was also significant [*F*(1,34) = 15.36, *p* < 0.001, η^2^ = 0.31], and vMMN was more negative in the fronto-central regions than the occipito-temporal.

With regard to late vMMN values (370–450 ms), the main effect of Expression condition remained significant [*F*(1,34) = 3.98, *p* = 0.05, η^2^ = 0.11], wherein vMMN was greater in the fearful condition than the happy condition. Anterior-posterior distribution was also significant [*F*(1,34) = 23.05, *p* < 0.001, η^2^ = 0.4], and vMMN was more negative in the fronto-cental regions relative to the occipito-temporal. The interaction between Expression × Age group was significant [*F*(1,34) = 3.98, *p* = 0.06, η^2^ = 0.11], and the *post hoc* analyses indicated that adults featured a more negative vMMN in the fearful condition relative to the happy condition (*p* < 0.05).

## Discussion

The current study investigated the neurodevelopmental processes underlying the automatic detection of changes in facial expression. This was achieved by evaluating the electrophysiological responses of adolescents and adults to emotional and neutral facial stimuli. The main findings of this study indicated that adolescents featured a greater level of vMMN compared to adults in the fronto-central areas in early stage of processing, and only adults featured a more negative vMMN in the fearful condition relative to the happy condition in the late stage of processing.

### Sensory Responses

Changes in visual evoked potentials (VEPs) are reported to persist throughout late childhood and adolescence, likely due to the continuous electrophysiological maturation of the human visual system (Brecelj et al., 2002; Clery et al., 2012). With regard to the ERP responses evoked by deviant and standard stimuli, adolescents demonstrated larger P1 amplitudes, indicating that adolescents featured stronger early visual detection processes on facial expressions than adults (Guyer et al., 2008; Passarotti et al., 2009; Scherf et al., 2012; van den Bulk et al., 2012). The N170 component reflects the structural encoding of facial expression (Bentin et al., 1996), and shorter N170 latencies relate to more accurate facial perception in both adolescents and adults (McPartland et al., 2004). In the present study, adults demonstrated greater N170 amplitudes and faster responses, supporting reports of more accurate structural encoding in adults versus adolescents. These findings were consistent with previous reports that adult N170 response patterns could not be reached even by the mid-adolescence and late-adolescence (Taylor et al., 1999, 2004; Itier and Taylor, 2004a,b; Batty and Taylor, 2006; Miki et al., 2011). Moreover, with regard to the LPP component, adults exhibited faster LPP responses in the fearful condition than that in the happy condition. This is in agreement with the prevalence of negativity bias and suggests that a negative stimulus triggers a greater degree of context evaluation and stimulus elaboration than a positive stimulus (Williams et al., 2006).

### Visual Mismatch Responses

Visual mismatch negativity is reported to reflect the automatic process underlying the detection of mismatches between sensory input and the representation of frequently presented stimuli in transient memory (Czigler, 2007). vMMN relates to prediction errors arising between the transient representation of visual information and actual perceptual input (Winkler and Czigler, 2012; Stefanics et al., 2014, 2015). Sources of vMMN are reported to include retinotopic regions of the visual cortex (Czigler et al., 2004; Sulykos and Czigler, 2011) and prefrontal regions (Kimura et al., 2010, 2011). Despite an increasing number of vMMN studies in adults, relatively few have examined vMMN in children and adolescents. Clery et al. (2012) examined the visual mismatch response (vMMR) in children (8–14 years) and adults. In particular, the authors reported that the detection of changes in non-emotional information required a longer duration for children in late childhood than adults (Clery et al., 2012). In addition, Tomio et al. (2012) investigated the developmental changes in vMMN for subjects aged 2–27 years old, and observed that vMMN latencies decreased with age, maturing to adult level by the age of 16 years. In contrast, another ERP study reported no significant differences in vMMN responses (180–400 ms) to color modality between children (aged ∼10 years) and adults (Horimoto et al., 2002).

The current study was the first to investigate vMMN with regard to the development of emotional processing in adolescence. In this experiment, adolescents demonstrated a greater level of vMMN compared to adults in the fronto-central regions during the early processing phase (120–200 ms), which might indicate that adolescents are more sensitive to changes in facial expression at this stage. This early vMMN finding correlated with P1 amplitude (peaking during 100–170 ms), wherein adolescents featured greater P1 amplitudes compared to adults. Since this early vMMN overlaps with the P1 time window, it is possible to suggest that early vMMN represents the initial processing of standard and deviant stimuli reflected by P1 amplitude. Furthermore, adolescents featured more negative vMMN responses in the fronto-central regions than the occipito-temporal, which was not observed in adults. This was consistent with previous studies, in which enhanced prefrontal activity was identified in adolescents in response to affective faces (Killgore et al., 2001; Yurgelun-Todd and Killgore, 2006). Several frontal structures are considered to be essential neural interfaces between cognitive and emotional processes, including the ventrolateral prefrontal cortex (VLPFC) and dorsal anterior cingulated cortex (dACC) are (Petrides and Pandya, 2002; Pavuluri et al., 2007). Furthermore, the frontal cortex undergoes a prominent transformation during adolescence (Gogtay et al., 2004; Taylor et al., 2014), which correlates strongly with the profound neural development of social-emotional processing during this period (Monk et al., 2003; Palermo and Rhodes, 2007; Blakemore, 2008; Fusar-Poli et al., 2009).

Adults showed more negative late vMMN (370–450 ms) in the fearful condition than the happy condition, which was consistent with previous expression-related vMMN studies (Kimura et al., 2011; Stefanics et al., 2012). This also highlighted the enhanced automatic detection of fearful expressions relative to happy, which is in line with the effects of negativity bias, wherein individuals demonstrate comparatively faster behavioral responding and/or greater ERP responses to negative stimuli compared to positive or neutral stimuli (Nelson and de Haan, 1996; Ito et al., 1998; Carretié et al., 2001; Batty and Taylor, 2003; Smith et al., 2003; Dawson et al., 2004; Rossignol et al., 2005; Leppänen et al., 2007; Vaish et al., 2008). However, adolescents did not show such differences, and they had comparable vMMN responses in fearful condition and happy condition, which suggested that during late automatic processing phase adolescents’ neural automatic processing to both types of affective information were equal.

With regard to vMMN during the middle and late processing phases (230–320 ms and 370–450 ms, respectively), both groups demonstrated greater vMMN responses in the fronto-central regions than the occipito-temporal. This indicates that fronto-central brain regions were strongly activated in response to affective changes in both adults and adolescents. Accordingly, mismatch components with regard to the processing of emotional faces have also been reported in the frontal regions (Astikainen and Hietanen, 2009; Kimura et al., 2012; Stefanics et al., 2012; Astikainen et al., 2013), wherein Kimura et al. (2012) demonstrated that frontal activation was observed in all phases of vMMN. In addition, Yurgelun-Todd and Killgore (2006) reported that individual’s age was positively correlated with stronger neural activation in the prefrontal cortex during facial perception with regard to fearful but not happy stimuli in children and adolescents. The neural generators of vMMN with regard to non-emotional information have also been observed in the medial prefrontal and lateral prefrontal areas (Yucel et al., 2007; Kimura et al., 2010; Urakawa et al., 2010). Moreover, prior neuroimaging studies have reported that adolescents feature greater amygdala and fusiform activation during passive viewing of fearful expressions (Guyer et al., 2008). Therefore, the effects of negativity bias in adolescents indicate that the recognition of negative expressions is essential for social-emotional development (Williams et al., 2009).

Two limitations were identified within the current study: first, no vMMN data on non-emotional stimuli was collected in adolescents, hence, it was uncertain that the present observed vMMN effects between adolescents and adults were on facial emotion specificity. In further studies, a control condition could be included with a non-emotional standard and deviant, in order to analyze both emotional and non-emotional vMMN in both adolescents and adults. Second, a flip-flop design, as used by Stefanics et al. (2012), was not implemented in the current study. In a flip-flop paradigm, the images used as the standard and deviant visual stimuli are subsequently reversed in the oddball sequence. In future work, incorporating a flip-flop design would enable the analysis and comparison of vMMN responses induced by reversal of the oddball design.

## Conclusion

The current study investigated the neurodevelopmental processes underlying the automatic detection of changes in emotional expression in adolescents. Adolescents demonstrated stronger automatic processes with regard to the perception of emotional faces in early processing stage. During the late phase, only adults showed better automatic processing on fearful faces compared to happy faces, while, adolescents had comparable automatic change processes on fearful and happy expressions. The present findings revealed dynamic differences in the automatic neural processing of facial expression between adolescents and adults.

## Author Contributions

TL designed the experiment and collected the data. TX analyzed the data. TL, TX, and JS wrote the MS.

## Conflict of Interest Statement

The authors declare that the research was conducted in the absence of any commercial or financial relationships that could be construed as a potential conflict of interest.
